# Losartan Reverses Hippocampal Increase of Kynurenic Acid in Type 1 Diabetic Rats: A Novel Procognitive Aspect of Sartan Action

**DOI:** 10.1155/2019/4957879

**Published:** 2019-10-13

**Authors:** Iwona Chmiel-Perzyńska, Adam Perzyński, Bartosz Olajossy, Paulina Gil-Kulik, Janusz Kocki, Ewa M. Urbańska

**Affiliations:** ^1^Department of Experimental and Clinical Pharmacology, Medical University in Lublin, Poland; ^2^II Department of Psychiatry and Psychiatry Rehabilitation, Medical University in Lublin, Poland; ^3^Internal Medicine and Cardiology Clinic, 1st Military Clinical Hospital in Lublin, Poland; ^4^Department of Clinical Genetics, Medical University in Lublin, Poland; ^5^Laboratory of Cellular and Molecular Pharmacology, Department of Experimental and Clinical Pharmacology, Medical University in Lublin, Poland

## Abstract

Patients with diabetes mellitus (DM) type 1 and 2 are at a higher risk of cognitive decline and dementia; however, the underlying pathology is poorly understood. Kynurenic acid (KYNA), endogenous kynurenine metabolite, displays pleiotropic effects, including a blockade of glutamatergic and cholinergic receptors. Apart from well-known glial origin, kynurenic acid is robustly synthesized in the endothelium and its serum levels correlate with homocysteine, a risk factor for cognitive decline. Studies in an experimental DM model suggest that a selective, hippocampal increase of the kynurenic acid level may be an important factor contributing to diabetes-related cognitive impairment. The aim of this study was to assess the effects of chronic, four-week administration of losartan, angiotensin receptor blocker (ARB), on the brain KYNA in diabetic rats. Chromatographic and rt-PCR techniques were used to measure the level of KYNA and the expression of genes encoding kynurenine aminotransferases, KYNA biosynthetic enzymes, in the hippocampi of rats with streptozotocin-induced DM, treated with losartan. The effect of losartan on KYNA synthesis *de novo* was also evaluated *in vitro*, in brain cortical slices. The hippocampal increase of KYNA content occurred in diabetic rats treated and nontreated with insulin. Losartan did not affect KYNA levels when administered *per se* to naïve or diabetic animals but normalized KYNA content in diabetic rats receiving concomitantly insulin. The expression of *CCBL1* (*kat 1*), *AADAT* (*kat 2*), and *KAT3* (*kat 3*) genes did not differ between analyzed groups. Low concentrations of losartan did not affect KYNA production *in vitro*. The neuroprotective effect of ARBs in diabetic individuals may be, at least partially, linked to modulation of KYNA metabolism. The ability of ARB to modulate synthesis of KYNA in diabetic brain does not seem to result from changed expression of genes encoding KATs. We propose possible involvement of angiotensin AT_4_ receptors in the observed action of losartan.

## 1. Introduction

A dramatic increase of diabetes mellitus (DM) prevalence in human population presents a major challenge for clinicians. Despite remarkable improvement in therapy aimed to control glycaemia, gradually developing long-term complications of DM are the major causes of morbidity and mortality in DM type 1 and type 2 (DM1 and DM2) [1]. The incidence of central nervous system-related complications increases with the duration of disease, independently of the type of DM, and affects individuals with insulin dependent [1]. Patients with DM are at a higher risk of developing mild to moderate slowing of mental speed, decline of attention, diminished cognitive flexibility, and dementia [2]. DM-associated cognitive decline has been linked primarily with the presence of microvascular complications and only partially with the occurrence of hypoglycemic episodes or with poor metabolic control [2]. However, the precise nature of events leading to cognitive impairment in the course of DM is poorly understood.

Kynurenic acid (KYNA) is the only known neuroprotective derivative of tryptophan, produced in the brain and periphery along the kynurenine pathway, in the process of enzymatic conversion of L-kynurenine by kynurenine aminotransferases (KATs) [3]. Synthesis of KYNA is regulated by various endo- and exogenous factors including extracellular ions, presence of sulphur-containing amino acids, or exposure to environmental toxins [4–6]. Functionally, KYNA is a pleiotropic molecule blocking the activation of glutamate receptors and *α*_7_ nicotinic receptors and acting as a ligand of GPR35 and AHR receptors [3, 7]. Neuroprotective effects of KYNA were confirmed under *in vitro* and *in vivo* conditions, in various models of excitotoxicity, including, e.g., ischemia, anoxia, or seizures [3, 8]. Conversely, considering the essential role of glutamate-mediated neurotransmission in learning and memory processes, excessive formation of KYNA was implicated in cognitive decline, and increased KYNA content in selected cortical areas was reported in patients with schizophrenia [9, 10]. Interestingly, serum KYNA was found to correlate with the level of homocysteine in stroke patients [11]. Considering that hyperhomocysteinemia is recognized as a modifiable risk factor for the development of cognitive decline and dementia, these data further support possible contribution of KYNA to cognitive decline.

Following the discovery that vascular endothelium is an important source of circulating KYNA [12], clinical data connecting the disturbed KYNA production with vascular dysfunction have followed [13]. In the view of reports indicating that endothelial dysfunction is a critical factor in the genesis of diabetic vascular complications, we focused on the interplay between KYNA and DM, using the experimental *in vitro* and *in vivo* approach. In cortical slices, hyperglycemia potentiated the inhibitory effect of mitochondrial toxins and D,L-homocysteine on KYNA synthesis, whereas ketone body, *β*-hydroxybutyrate, increased KYNA production in a protein kinase A-dependent way, most probably *via* stimulation of KAT activity [4, 14]. *In vivo*, streptozotocin- (STZ-) induced DM in rats evoked a selective increase of the hippocampal KYNA level, an effect that was not reversed by insulin treatment [15]. Abnormal function of the hippocampus, crucial for proper learning and memory formation, is one of the hallmarks of diabetic cognitive impairment [16]. Thus, high KYNA levels in this brain area may contribute to the deterioration of cognition.

The role of angiotensin-converting enzyme inhibitors (ACE-I) and antagonists of ANGII receptors (ARBs) in the therapy and/or prevention of cognitive decline has gained prominent interest within the last decade [17]. ANGII, via interaction with two major types of receptors, AT_1_ and AT_2_, present on neurons, cerebrovascular endothelial cells, and astrocytes, affect synaptogenesis, neuronal migration, and cerebral blood flow regulation, as well as in memory and cognitive processes [18, 19]. Excessive stimulation of AT_1_ receptors may initiate inflammatory response via a cascade of events including synthesis/activation of proinflammatory cytokines and enzymes and was suggested as one of the crucial factors contributing to the development of peripheral and central diabetic complications [20, 21]. Studies on drugs modulating a renin-angiotensin system in patients with dementia have yielded promising results [22]. The onset of disease seems to be delayed, and an incidence of cognitive decline is reduced during therapy with ARBs [23]. However, the precise link of events underlying the beneficial influence of ARBs on cognitive processes is not fully recognized. Considering observations from our laboratory on the inhibitory action of ARBs on KYNA synthesis *in vitro*, in endothelial and cortical slices [24, 25], we hypothesized that losartan (LOS) may selectively reverse DM-induced changes in hippocampal KYNA synthesis *in vivo*. The level of KYNA as well as the expression of genes encoding KYNA biosynthetic enzymes was assessed in the hippocampi of rats with experimentally induced DM1 and chronically treated with LOS.

## 2. Materials and Methods

### 2.1. Animals

Experiments were performed on male Wistar rats, weighing initially 200-220 g. Animals were housed under standard laboratory conditions (18°C environmental temperature; 12 h light/dark cycle, with food and water available *ad libitum*). All animal handling and experimental procedures were performed in accordance with the EC (EEC Council Directive 86/609 1987) and have been approved by the Local Ethical Committee in Lublin. All efforts were made to minimize animal suffering and to reduce the number of animals used.

### 2.2. Substances

Streptozotocin (STZ) and L-kynurenine sulphate salt were obtained from Sigma-Aldrich (St. Louis, U.S.A.). Neutral protamine Hagedorn (NPH) insulin (Gensulin N) was received from Bioton, and 0.9% NaCl was acquired from Polpharma. LOS (Xartan) was obtained from Adamed. All of the high-pressure liquid chromatography (HPLC) reagents were purchased from J.T. Baker Laboratory Chemicals (Holland). Other reagents were obtained from POCH (Gliwice, Poland).

### 2.3. In Vitro Studies

Synthesis of KYNA in rat brain cortical slices was performed as described before [26]. After decapitation, rat brains were immediately removed from the skull. The cerebral cortex was harvested and cut into slices (1 × 1 mm) using the McIlwain tissue chopper. Slices were placed randomly into culture wells (10 slices per well; 24-well plates) containing Krebs-Ringer buffer (KRB), pH 7.4, composed as follows: 118.5 mM NaCl, 4.75 mM KCl, 1.77 mM CaCl_2_, 1.18 mM MgSO_4_, 12.9 mM NaH_2_PO_4_, 3 mM Na_2_HPO_4_, and 5 mM glucose (final concentrations). KRB was oxygenated for 30 min (95% O_2_, 5% CO_2_), prior to experiment. After preincubation period (15 min, 37°C), LOS was added to incubation media. Incubation was initiated by the addition of L-kynurenine (final concentration 10 *μ*M) and carried out for 2 hours at 37°C. Then, media were separated from the tissue and placed at ice bath. Subsequently, media were mixed with 0.1 ml of 1 N HCl and 14 *μ*l of 50% trichloroacetic acid and centrifuged. Supernatants were applied to cation-exchange columns containing Dowex 50 W^+^ (200-400 mesh; Sigma-Aldrich) prewashed with 1 ml of water and 1 ml of 0.1 M HCl. Columns were subsequently washed with 1 ml of 0.1 N HCl and 1 ml of water, and KYNA was eluted with 2.5 ml of water. At least 6 wells were used for each concentration of studied drug, and each experiment was repeated at least twice for each concentration. Blanks, containing all solution but without brain tissue, were prepared along each experiment. KYNA was quantified fluorimetrically, as described below.

### 2.4. In Vivo Studies

#### 2.4.1. Experimental Protocol

Rats were randomly assigned to one of the 6 following groups: (A) control group (CTR), nondiabetic, healthy rats receiving vehicle; (B) LOS, nondiabetic rats receiving LOS; (C) DM, diabetic rats; (D) DM+LOS, diabetic rats receiving LOS; (E) DM+INS, diabetic rats receiving insulin; and (F) DM+INS+LOS, diabetic rats receiving insulin and LOS. Experimental groups initially included 12 animals each.

DM1 was induced by single intraperitoneal (i.p.) administration of STZ, diluted in 0.05 M citrate buffer, pH 4.2, in the dose of 60 mg/kg [15]. CTR was given appropriate volume of 0.05 M citrate buffer instead of STZ. Administration of insulin was initiated after confirmation of glucosuria (Ketodiastix-Bayer), one week after the injection of STZ. NPH insulin was given once daily, between 9:00 and 10:00 a.m., subcutaneously (s.c.), in the substitution dose of 9 IU/kg [27], sufficient to prevent glucosuria. Groups A-D received physiological saline i.p., instead of insulin. LOS was dissolved in sterile water and injected i.p. at the dose of 20 mg/kg, between 9:00 and 10:00 a.m., starting on the same day as insulin therapy. Treatment was conducted for four weeks. All animals were monitored daily for body weight, and diabetic animals (C-F) were monitored daily for glucosuria and ketonuria.

#### 2.4.2. Measurements of KYNA Content in the Hippocampus

Animals were sacrificed through decapitation 35 days after STZ administration. Brains were quickly removed, chilled, rapidly dissected, and stored at -72°C. On the day of analysis, brain samples were homogenized (sonicator; Bandelin Sonopuls) 1 : 10 (weight : volume) in distilled water. Homogenate was centrifuged (13600xRCF, 5 minutes, 4°C), acidified (0.1 ml of 1 N HCL and 14 *μ*l of 50% trichloroacetic acid), and centrifuged again. Supernatants were applied to the cation-exchange columns (Dowex 50 W^+^, 200-400 mesh), prewashed with 1 ml of water and 1 ml of 0.1 N HCl. Columns were subsequently washed with 1 ml of 0.1 N HCl and 1 ml of water. KYNA was eluted with 2.5 ml of water.

#### 2.4.3. Quantification of KYNA

Eluted KYNA was subjected to the HPLC and quantified fluorimetrically (Varian HPLC system; ESA catecholamine HR-80.3 *μ*m, C_18_ reverse-phase column), as previously described [26]. The mobile phase (pH 6.2) contained 250 mM zinc acetate, 50 mM sodium acetate, and 4% acetonitrile. Each chromatographic assay was preceded by the measurements of standardized concentrations of KYNA (0.2, 0.4, 0.6, 0.8, and 1.0 pmol), in order to obtain calibration curve.

### 2.5. Genetic Analyses

The expression of genes responsible for production of kynurenine pathway enzymes *CCBL1* (*Kat 1*), *AADAT* (*Kat 2*), and *KAT3* (*Kat 3*) was assessed as follows:

#### 2.5.1. Isolation of Total Cellular RNA

Total cellular RNA was isolated from the brain tissue using the method by Chomczynski and Sacchi [28], in modification. Brain samples were homogenized in 0.5 ml of TRI-reagent solution (Sigma-Aldrich, USA) and centrifuged for 10 minutes (4°C, 14000 rpm). After mixing with chloroform (Sigma-Aldrich, USA), the supernatant (0.125 ml) was thoroughly mixed, incubated for 15 minutes (room temperature), and again centrifuged (4°C, 14000 rpm). The liquid phase was separated, isopropanol (Sigma-Aldrich, USA) was added (0.25 ml), and the mixture was centrifuged for 20 minutes, first at room temperature and then at 4°C (20 minutes, 14000 rpm). The supernatant was discarded. Two ml of 75% ethanol (0.2 ml) was added to RNA precipitate and centrifuged (4°C, 14000 rpm). Precipitate was diluted with RNase-free ultrapure water (Baker). RNA extract purity and concentration were evaluated with a spectrophotometric method with NanoDrop 2000c equipment (ThermoFisher Scientific, USA).

#### 2.5.2. cDNA Synthesis and Reverse Transcription Reaction

Reverse transcription (RT) was performed with the set of reagents High-Capacity cDNA Transcription Kits with RNase Inhibitor (Applied Biosystems, USA). The reaction was conducted in 20 *μ*l volume, according to the manufacturer's instructions. Each reaction mixture contained 1 *μ*g of isolated RNA diluted in 10 *μ*l of RNase-free ultrapure water, 2 *μ*l of 10xRT Buffer, 2 *μ*l of 10xRT Random Primer, 0.8 *μ*l of 10xdNTPs (100 mM), 1 *μ*l of RNase 20 U/*μ*l, 1 *μ*l of reverse transcriptase (50 U/*μ*l), and 3.2 *μ*l of ultrapure water. The cDNA was synthesized on Veriti Dx (Applied Biosystems, USA) under the following conditions: stage I: 25°C, 10 min; stage II: 37°C, 120 min; stage III: 85°C, 5 min.

#### 2.5.3. PCR in Real Time

Polymerase chain reaction in real time (rt-PCR) was conducted with StepOnePlus System (Applied Biosystems, USA) in 96-well plates, in the volume of 25 *μ*l. The mixture contained 1 *μ*l of cDNA after reverse transcription reaction, 10.25 *μ*l of RNase- and DNase-free ultrapure water, 12.5 *μ*l of Gene Expression Master Mix (Applied Biosystems, USA), and 1.25 *μ*l of specific for study gene probe. The following molecular probes were used: for *Aadat* (*Kat1*), Rn00567882_m1; for *Ccbl1* (*Kat2*), Rn01439191_m1; for *Kat3* (*Kat 3*), Rn01522582_m1; and for *Gapdh*, Rn01775763_g1. The expression of Gapdh served as endogenous CTR. The reaction was conducted in the following cycles: initial denaturation, 10 minutes in 95°C, next 40 cycles—the first stage: 15 seconds in 95°C, the second stage: 60 seconds in 60°C. The relative gene expression (RQ) in each studied sample with reference to CTR sample was calculated with the following formula: RQ = 2^−ΔΔCt^ (*Livak 2001*).

### 2.6. Statistical Analyses

The expression of the following genes: *Aadat* (*Kat 1*), *Ccbl1* (*Kat 2*), and *Kat3*, normalized with the reference to Gapdh, was analyzed with Expression Suite Software v1.0.3 (ThermoFisher Scientific). The statistical analyses of data concerning glucose and KYNA levels were performed using the one-way ANOVA test, with the adjustment of *p* value by the Bonferroni method. Data are presented as mean values ± SD.

## 3. Results

### 3.1. *In Vitro* Studies

**Figure 1:**
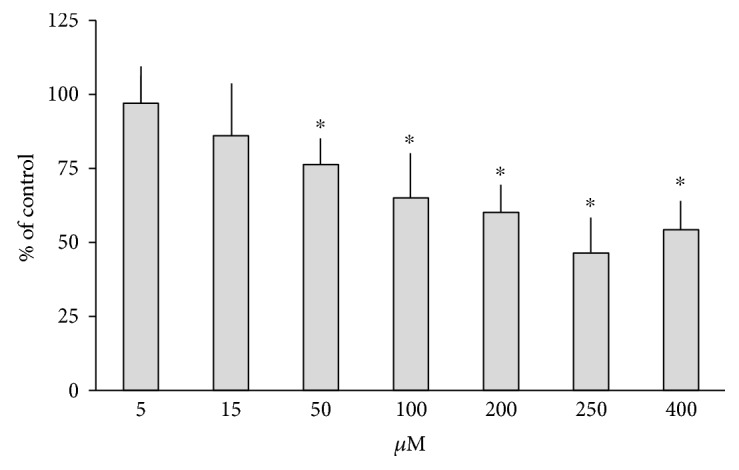
The influence of losartan (LOS) on the kynurenic acid (KYNA) production in rat brain cortical slices. The data are presented as a percentage of control values. ^∗^*p* < 0.05 vs. control (ANOVA with the adjustment of *p* value by the Bonferroni method).

LOS at 50-400 *μ*M, but not at 5 and 15 *μ*M concentration, significantly decreased *de novo* synthesis of KYNA in rat brain cortical slices (Figure 1).

### 3.2. *In Vivo* Studies

Chronic administration of LOS has not changed the serum glucose level in the experimental animal level, in comparison with CTR (145 ± 22 vs. 138 ± 31 mg/dl; ns). In the DM group, glycaemia was significantly higher and reached 563 ± 83 mg/dl (408% of CTR; *p* < 0.001). Similarly, in the DM+LOS group, serum glucose was significantly higher than in CTR and reached 505 ± 150 mg/dl (366% of CTR; *p* < 0.001). Treatment with insulin normalized glycaemia in the DM+INS group to 151 ± 35 mg/dl (110% of CTR; ns) and in the DM+INS+LOS group to 172 ± 29 mg/dl (124% of CTR; ns).

### 3.3. KYNA Content in the Hippocampus

**Figure 2:**
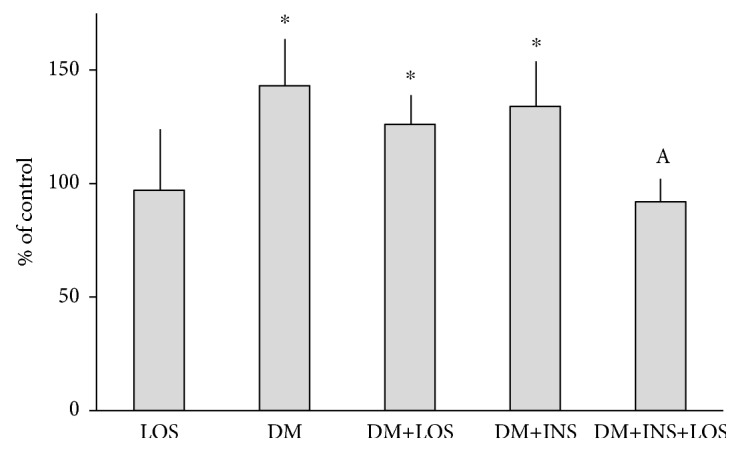
The effect of losartan on a hippocampal increase of KYNA in diabetic rats. Values are arithmetic means ± SD; ^∗∗^*p* < 0.01 vs. CTR; ^a^*p* < 0.05 vs. respective DM (ANOVA with the adjustment of *p* value by the Bonferroni method). CTR: nondiabetic, healthy rats receiving vehicle; LOS: nondiabetic rats receiving losartan; DM: animals with streptozotocin- (STZ-) induced diabetes; DM+LOS: diabetic animals treated with losartan for 28 days; DM+INS: diabetic animals treated with insulin for 28 days; DM+INS+LOS: diabetic animals treated with insulin and losartan for 28 days.

There was a significant increase of KYNA content in diabetic animals treated and nontreated with insulin (DM and DM+INS) in comparison with CTR (143 and 137% of CTR, respectively; both *p* < 0.05 vs. CTR) (Figure 2). Chronic administration of LOS prevented the increase of KYNA content in diabetic animals treated with insulin (DM+INS+LOS) (92% of CTR; ns vs. CTR) but not in diabetic animals not treated with insulin (DM+LOS) (124% of CTR, ns vs. CTR) (Figure 2). A 4-week therapy with LOS itself has not significantly affected the level of KYNA in the hippocampus (Figure 2).

### 3.4. Gene Expression

**Figure 3:**
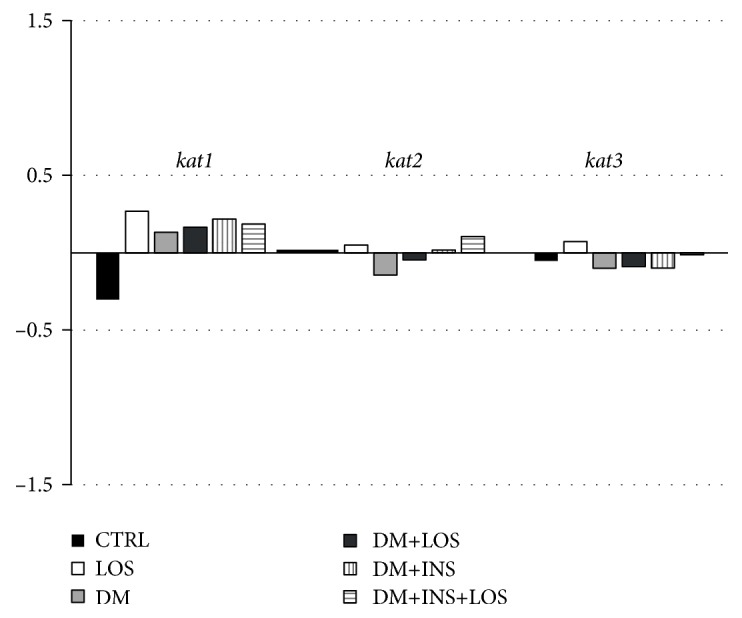
The influence of losartan on the hippocampal expression of genes encoding KAT I, KAT II, and KAT III (*CCBL1* (*kat 1*), *AADAT* (*kat 2*), and *KAT3* (*kat 3*)) in diabetic rats. Normalized with the reference to Gapdh expression of genes was analyzed with Expression Suite Software v1.0.3 (ThermoFisher Scientific). Data are presented as LogRQ. CTR: nondiabetic, healthy rats receiving vehicle; LOS: nondiabetic rats receiving losartan; DM: animals with streptozotocin- (STZ-) induced diabetes; DM+LOS: diabetic animals treated with losartan for 28 days; DM+INS: diabetic animals treated with insulin for 28 days; DM+INS+LOS: diabetic animals treated with insulin and losartan for 28 days.

There were no statistically significant differences in the expression of examined genes, *CCBL1* (*kat 1*), *AADAT* (*kat 2*), and *KAT3* (*kat 3*), between analyzed groups in the hippocampi of animals with DM, DM+INS, DM+LOS, DM+INS+LOS, or LOS (Figure 3).

## 4. Discussion

Presented data indicate that LOS, commonly used in medical practice ARB, prevents experimental DM-induced increase of the hippocampal KYNA level when coadministered with insulin. LOS did not influence the DM-evoked changes in KYNA levels when given to diabetic animals not treated with insulin. Furthermore, in healthy CTR animals, LOS did not change the hippocampal KYNA level. These observations suggest that ARBs target cellular mechanisms initiated by the development of DM and act synergistically with insulin. *In vitro*, LOS reduced KYNA synthesis already at micromolar concentrations, as shown before.

Based on the fact that LOS can easily cross the blood-brain barrier [29] and that modulation of KYNA levels did not occur in healthy animals, we may exclude peripheral cardiovascular changes as the mechanism underlying observed effect. Data obtained *in vitro* in our laboratory [24, 25] show that ARBs display specific, inhibitory effects on KYNA synthesis associated with inhibition of KAT II activity. In here, significant inhibitory action was observed in cortical slices treated with LOS of 50 *μ*M and higher concentrations. It is conceivable to assume that LOS given peripherally should reach brain compartment in levels high enough to affect KYNA synthesis. Indeed, normalization of DM-evoked perturbations of KYNA synthesis has been observed in the hippocampus, known for its crucial role in learning and memory processes. However, the production of KYNA remained unaltered in the brains of healthy animals treated with LOS, and literature data indicate that the serum level of LOS in animals treated with analogical dose of LOS is below 1 *μ*M [30]. Therefore, it cannot be excluded that under *in vivo* conditions, mechanisms other than inhibition of KYNA biosynthetic enzymes may contribute to alterations of KYNA production.

The expression of genes encoding enzymes responsible for synthesis of KYNA, *CCBL1* (*kat 1*), *AADAT* (*kat 2*), and *KAT3* (*kat 3*), was not altered in the brains of experimental animals, which argues against transcriptional regulation of hippocampal KYNA in this scenario.

Broad array of diabetes-related changes in the hippocampal region involves altered neuronal morphology, disturbed axonal transport, and modifications of synaptic proteins, followed by spatial learning and memory deficits [16]. Cell surface G-protein-coupled AT_1_ receptors are the major targets of ANGII within the brain. Upon their activation, the long-term potentiation (LTP) process is compromised, and learning and memory acquisition are impaired. Furthermore, stimulation of AT_1_ receptors may result in the excessive release of proinflammatory cytokines and free radicals and increased production of *β*-amyloid [20, 21, 31]. Antagonists of AT_1_ receptors are promising candidates in the therapy of various neuropathological conditions, such as stroke, neurodegenerative disorder or traumatic brain injury or mood disorders, and DM-related cognitive impairment [32]. Indeed, in various animal models of Alzheimer's disease and stroke, ARBs reduce cerebrovascular, neuropathological, and cognitive deficits [33, 34]. Reduction of AT1-initiated oxidative stress, as well as upregulation of pathways mitigating neuronal death, including an increased expression of neurotrophic factors and reduction in ER stress markers, seems to underlie protective action of ARBs [33, 34]. Retrospective clinical analysis showed that ARBs reduce the risk of Alzheimer's disease among hypertensive patients [35].

Noteworthy, ARBs display also anti-inflammatory effects, under *in vitro* and *in vivo* conditions. Telmisartan reduced inflammatory changes in the cortex and hippocampus evoked by transient middle cerebral artery occlusion, without lowering blood pressure [36]. LOS, at *μ*M concentration, suppressed the LPS stimulation of microglial cell cultures, preventing increases of NF-kappaB and activator protein-1 activity [37], and protected against ischemia-induced apoptosis *in vitro*, in an Akt-phosphorylation-dependent way [38]. Candesartan exerted anti-inflammatory action in cultured rat microglia, cerebellar granule cells, and cerebral microvascular endothelial cells exposed to LPS [20]. Furthermore, ARBs activate the peroxisome proliferator-activated receptor (PPAR-*γ*), a member of the nuclear receptor superfamily of ligand-activated transcription factors, playing an important role in lipid and glucose homeostasis. However, the effect was not reversed by PPAR-*γ* antagonist and seems to be rather weak *in vivo* [39, 40].

Novel observations suggest that apart from a blockade of AT_1_ receptors, effects of LOS may be actually linked to an activation of AT_4_ receptors, stimulated physiologically by ANGIV [41]. The AT_4_ receptor was identified as mediating the cognitive and cerebrovascular beneficial of LOS in a mouse model of Alzheimer's disease, and the AT_4_ receptor-mediated effects were independent from changes in blood pressure, amyloidosis, and oxidative stress [42]. Central AT_4_ receptors are broadly distributed in various brain areas, with very high density in the hippocampus, and are implicated in the regulation of exploratory behavior as well as learning and memory acquisition [42]. Convincing evidence suggests that receptor for ANGIV is identical with membrane-bound insulin-regulated aminopeptidase (IRAP) and colocalized with glucose transporter 4 (GLUT4) [43]. Targeting hippocampal IRAP is emerging as a novel therapy of learning and memory disturbances [44, 45]. ANGIV is thought to facilitate learning and memory by blocking the catalytic site of IRAP which may result in altered levels of neuropeptides or improved neuronal glucose uptake [44, 45]. It is tempting to hypothesize that activation of AT_4_ receptors by ARBs requiring simultaneous presence of sufficient amounts of insulin underlies the regulatory effect of LOS on KYNA synthesis in diabetic brain. Further research should be aimed at verifying this hypothesis.

In summary, we report that in the rodent model of DM, the chronic therapy with insulin and LOS restores the hippocampal KYNA level to control values. The above data reveal a novel mechanism which may contribute to beneficial effects of ARBs on cognitive processes.

## Data Availability

All the data were included in the submitted manuscript.

## Abbreviations

DM: Diabetes mellitus
KYNA: Kynurenic acid
KAT: Kynurenine aminotransferase
ANG II: Angiotensin II
AT_1_ receptor: Angiotensin type 1 receptor
AT_2_ receptor: Angiotensin type 2 receptor
ACE-I: Angiotensin-converting enzyme inhibitor
ARB: Angiotensin receptor blocker
LOS: Losartan
STZ: Streptozotocin
IRAP: Insulin-regulated aminopeptidase.
